# Monthly Summary

**Published:** 1875-05

**Authors:** 


### MONTHLY SUMMARY.

---

*Death from Chloroform.*—A young lad, aged 14, died on the 15 inst., at the Royal Free Hospital, whilst under the influence of chloroform. Mr. F. J. Gant, surgeon, said, that when the deceased was admitted, it was thought that the hip had been dislocated. When he examined the lad, it gave him great pain. He ordered Mr. Thompson, house-surgeon, to administer chloroform. The chloroform was administered for about two minutes, during which time about sixty drops were inhaled The deceased seemed to be half sensible; and on looking round, they saw the lad fainting, and in a dangerous state. Silvester's respiratory method and galvanism were applied, but without effect, for the lad died at once. A *post-mortem* examination was made. All the organs were sound. The cause of death was syncope from the effects of chloroform. A verdict of death from misadventure was returned.—*British Medical Journal.*

---

*On Right-Handedness*—This was the subject of a discussion at the Cambridge Philosophical Society last November. Dr. W. Ainslie Hollis presented a paper, in which right-handedness was attributed to the larger size of the left cerebral hemisphere, and its greater complexity and richness in gray matter. But this may be consequence rather than cause. Dr. Hillis claimed that right-handedness is peculiar to the human race, in which he is certainly wrong, (see Flint's *Physiology*, vol v, p. 457.) Some apes are left-handed, but most are right-handed. Lawson, in his "History of North Carolina," (1706,) makes the remark that the Indians are right-handed. (Is this true of all tribes?) Professor Humphries, a very competent anatomist, concluded the discussion by saying that he could see no anatomical reason for the preference of the right limb, the slight advantage in circulation to the right arm through the innominate artery and vein applying, in nearly equal degree, to the right side of the brain. He agreed that right-handedness was much a matter of education, and followed from the multifarious single-handed offices which are associated with the higher mental endowments. —*Med. and Surg. Reporter.*

*Detection of Falsification of Wax.*—The presence of resin in wax may be detected by its odor, or by treating the wax in the cold with alcohol ; the alcohol dissolves the resin, the wax being almost entirely or quite insoluble. The alcoholic solution evaporated to dryness gives the resin, which may be easily recognized.

On treating wax with oil of turpentine—*starch*, and earthy substances are left as a residue, the wax only being dissolved.

*Tallow* is recognized by its taste and disagreeable odor. Wax containing tallow is less brittle and greasy to the touch ; thrown upon live coals it produces thicker and darker smoke than pure wax.

To detect the presence of *stearine*, the wax is cut into small pieces, and boiled with lime-water ; if the wax is pure, the lime-water remains clear ; if, however, stearine is present, the lime-water becomes opaque, and deposits a white powder which is insoluble stearate of calcium.

Some manufacturers add considerable water to wax by agitation and fusion. This may be detected by the loss of weight of the wax when it is kept hot for some time upon the water bath.—*Druggists' Circular.*

---

*Death from Methylene.*—On Dec. 17, a death occurred at the Royal London Ophthalmic Hospital from the administration of bichloride of methylene. The patient was a woman aged twenty-five, suffering from firtula lachrymalis and caries of bone in the neighborhood of the lachrymal sac. A week previously the upper canaliculus had been laid open, together with a part of the outer wall of the sac, and a probe of large size was passed down the duct through an obstruction at its upper part. On that occasion the patient took methylene without any unfavourable symptom. On Tuesday two attempts were made by Mr. Couper to pass the probe, but as the patient seemed unable to endure the pain, it was thought advisable to have recourse to an anæsthetic. Bichloride of methylene was administered in the usual way by Mr. Buller, who for the last two years has been accustomed to administer this agent almost daily at the hospital, by means of a perforated leather inhaler covered with flannel. Three drachms by measure were poured into it (the ordinary quantity for an adult being four drachms.) At the end of about two minutes after the inhaler had been placed over the mouth and nose of the patient, her breathing suddenly became loud and stertorous. The anæsthetic was at once discontinued and the operation commenced. When the inhaler was removed the lips and cheeks were ruddy, but an unusual pallor of the alæ of the nose and skin around the mouth was noticed. The respiration, however, continued deep, full, and exaggerated. The inspirations were accompanied by loud palatal stertor, and the nostrils were observed to be flaccid, but there was no impediment to free access of the air to the lungs. Some seconds afterwards the pulse at the wrists rapidly failed, and then ceased almost suddenly, but the respiration continued for some time, and then failed rather suddenly. The tongue was immediately dragged forwards with forceps, and artificial respiration, by Silvester's method, established. The lower limbs and pelvis were at the same time gently raised from the couch, so as to favour gravitation of blood towards the brain. The face and breast were smartly slapped with a wet towel, and ammonia was applied to the nostrils. A strong solution of brandy and ammonia was thrown into the rectum as soon as possible, but was imperfectly retained owing to relaxation of the sphiniters. Artificial respiration was continued for forty minutes, but, with the exception of two or three sighing inspirations at intervals within the first few minutes, no sign of returning life was shown.—*Lancet.*

---

*On Poisoning by Carbolic Acid.*—Mr. F. Warren, resident surgeon at Steevens' Hospital, Dublin, relates (*Irish Hospital Gazette*, January 1) the case of a man who drank some solution of carbolic acid intended for disinfecting purposes, mistaking it for whiskey. Its action was most rapid; he immediately became insensible, falling down suddenly as in a fit; on his recovery he said that he remembered nothing whatever after tasting the liquid. When brought to the hospital he was suffering also from extreme syncope. The stomach-pump was used, stimulant enemata administered, and after about seven hours he recovered his consciousness and gradually rallied from the depression. An attack of acute gastritis followed. The urine passed the day after the accident was almost black, but was free from turbidity, and no trace of carbolic acid, blood, or albumen could be detected in it.—*London Med. Record.*

---

*Teeth and Oatmeal.*—Dr. Coles says, in the London *Medical Record:*

It has long been noted in this country that in those districts where the use of oatmeal (in place of wheaten flour) prevails, we find children and adults with the best developed teeth and jaws; and so well recognized is the influence of oatmeal diet upon the teeth, that many practitioners order its use as an article of daily diet for children, in cases where the dentition seems likely to be either retarded or imperfect.—*Med. & Surg. Reporter.*

*On the Relief of Pain by the External Use of Chloral.*—Dr. T. S. Dowse read before the Medical Society of London,( *Lancet*, Feb. 13, 1875,)a short peper on "the Relief of Pain by the external use of Chloral." He at first referred to the fact that until within the last few days he was not aware that Dr. Dujardin Beaumetz, of Paris, had largely experimented on the local use of this drug. He then showed that the local application of chloral will relieve pain, deaden sensibility, and allay reflex action when arising from irritation of the skin or mucous membrane. One of its most valuable qualities as a local application was its disinfecting power, and Dr. Dowse cited a number of cases of fungus hæmatodes, bed-sores, and sloughing wounds, in which it had acted as a more valuable disinfectant than anything else. Many other cases were also given illustrating the relief of pain produced by the local application of this drug, and Dr. Dowse also gave the means he adopted for its use. He uses four solutions—(1) a simple solution of four drachms of chloral to one pint of water, (2) one with glycerine, (3) one with perchloride of iron, and (4) one with chloride of zinc. Dr. Dowse concluded by remarking that the relief of pain by the external application of this agent was not effected by its absorption into the blood and by its direct action on sensory motor centres, but rather by immediate action on peripheral nerve terminations.—*Monthly Abstract of Med. Science.*

---

*Oleate of Mercury in Treatment of Ringworm.*—Dr. Cane ["London *Lancet*," November, 1873,] describes several cases in which he had used the oleate of mercury in treating ringworm. He says that over other remedies it has the following advantages: [1] It is a certain remedy if carefully applied. [2] It produces no staining or injury of the skin. This is of great importance where the disease appears on the face. [3] It is painless in its application, which is not true of the strong parasiticides. [4] It readily penetrates the sebaceous glands, hair follicles, and even into hairs themselves. Thus it is more likely to destroy the fungus than spirituous or aqueous solutions of mercury.—*Medical Weekly.*

---

*An Indigestible Morsel.*—At a late meeting of the Society Medicale des Hopitaux in Paris, M. Dujardin-Beaumetz exhibited a flattened leaden bullet which had been swallowed by a child. It had remained eight days in the digestive organs, produced asphyxia at the onset, and was voided naturally without having given rise to the least symptom of lead-poisoning, after treatment consisting of the administration of purgatives and sulphuric acid lemonade.—*London Med. Record.*

